# Editorial: Physiological adaptations of insects exposed to different stress conditions, volume II

**DOI:** 10.3389/fphys.2025.1582575

**Published:** 2025-03-18

**Authors:** Bimalendu B. Nath

**Affiliations:** ^1^ Stress Biology Research Laboratory, Department of Zoology, Savitribai Phule Pune University, Pune, India; ^2^ Faculty of Science, MIE-SPPU Institute of Higher Education, Doha, Qatar

**Keywords:** environmental stress, adaptation, insect, stressors, climate change

In the contemporary context of swiftly changing environmental conditions, non-human biota must adapt or risk extinction. Insects represent a significant portion of Earth’s biodiversity, with estimates of at least 5.5 million species (Stork, 2018). They are incredibly successful at adapting to extreme conditions. Numerous insect groups exhibit remarkable resilience, successfully evolving in response to varying environmental conditions. In recent years, more studies have gained insight into how insects will adapt to these stress pressures via physiological regulation. Recently, insect populations have faced continual stress from suboptimal temperature fluctuations due to global warming, seasonal shifts, moisture imbalances (such as desiccation), nutritional deficiencies stemming from habitat destruction (including deforestation and wildfires), and exposure to a broad range of xenobiotics (like pesticides and insecticides). Many insects have shown exceptional resilience to these multiple stressors, as evidenced by emerging physiological and genomic data reflecting phenotypic plasticity (Chakraborty et al., 2025; McCulloch and Jonathan, 2023). The ability of insects to adapt to various stressors results from complex physiological, genetic, behavioral, and ecological mechanisms. Ongoing research is essential to further understand these adaptations, especially in the context of rapid environmental changes. The adaptive capabilities of insects make them one of the most suitable animal models for examining survival strategies in response to natural and human-induced environmental selection pressures and their evolutionary processes.

The current Research Topic, titled “Physiological Adaptations of Insects Under Varied Stress Conditions,” serves as a continuation of the inaugural Research Topic published in Volume I (Tang et al., 2020). Recognizing an increasing interest among biologists focused on environmental stress responses regarding how insects adapt physiologically to different stressors, backed by significant genomic insights—the decision was made to develop this second volume in the series. We opted to broaden our exploration of various stress factors in the backdrop of ecological and evolutionary perspectives through two reviews and nine original research articles. Furthermore, this current volume represents an ongoing dialogue surrounding recent developments related to insect stress adaptation amidst diverse ecological challenges. The included reviews and research articles explore various adaptive traits through innovative protocols and emerging technologies aimed at filling existing knowledge gaps.

Under persistent selection pressure from chemical insecticides, insects have acquired resistance across various classes of these chemicals. Typically, the development of insecticide resistance within populations comes with a fitness cost that can influence the rate at which this resistance evolves. These fitness costs compromise biological characteristics such as survival rates, reproductive output, hatching success, and lifespan. This trade-off is viewed as indicative of the evolutionary processes driving insect resistance. Gul et al. extensively surveyed the literature on fitness costs induced by insecticide resistance published in the past decade. The review provides an in-depth analysis of these fitness costs, essential for formulating effective strategies to manage insecticide resistance.

The Manchineel, scientifically known as *Hippomane mancinella*—often called the “Death Apple Tree”—is recognized as one of the most poisonous fruits globally. Despite its toxicity, it is the exclusive host plant for the fruit fly species *A. acris*. García-Saldaña et al. described detoxification strategies employed by *Anastrepha acris* larvae. They identified two primary resistance mechanisms present in both species: a structural mechanism that activates cuticle protein biosynthesis—specifically chitin-binding proteins, which likely diminish permeability to harmful substances within the intestine—and a metabolic mechanism that induces serine protease production and enhances xenobiotic metabolism activation.

In recent years, the excessive use of synthetic insecticides has resulted in developing insect populations resistant to these chemicals. Consequently, an increasing focus is on advancing alternative eco-friendly insecticides that can contribute effectively to pest management strategies. Plumbagin has emerged as a significant compound within agricultural chemistry due to its unique mode of action and remarkable efficacy against a wide range of insects. The research conducted by Sun et al. shed light on the specific insecticidal mechanisms associated with plumbagin, offering important insights into its effectiveness as a natural pesticide.

As a promising and sustainable substitute for conventional pesticides, RNAi-based strategies facilitate the development of more eco-friendly and resilient agricultural practices to manage insect pests (Quilez-Molina et al., 2024). A noteworthy investigation by Yang et al. focused on trehalose, which serves as a key precursor in the chitin synthesis pathway within insects. The enzyme trehalose-6-phosphate synthase (TPS) plays an essential role in this biosynthetic process. Through RNA interference (RNAi), inhibiting TPS expression in *Mythimna separata* led to substantial reductions in both trehalose levels and TPS activity. Their findings suggest that RNAi technology may significantly enhance current approaches for controlling infestations of *M. separata*.

In light of the worldwide challenges posed by insect pests and the detrimental impacts of pesticide application, the sterile insect technique (SIT) was devised as early as the 1930s. This method for pest management is gaining increasing interest globally and applies to both minor and extensive operations. The SIT functions as an autocidal control strategy that necessitates large-scale breeding of specific target insects and sterilization through ionizing radiation, followed by systematic release into designated areas to decrease population numbers over successive generations. The spotted cutworm *Xestia c-nigrum* represents a significant pest in agroforestry across temperate and tropical climates in Asia, Europe, North Africa, and North America. Research conducted by Chu et al. focused on detailed dosimetry related to X-ray exposure and its effects on various biological parameters of this irradiated pest. Their results suggest that X-ray irradiation could effectively manage this versatile agricultural threat.

The melon fly, *Z. cucurbitae*, represents a significant insect pest affecting the Cucurbitaceae family globally, damaging over 130 varieties of vegetables and fruits. Previous discussions have highlighted the potential of radiation-mediated approaches as an alternative method for pest management that poses minimal risk to non-target insect species. In a separate investigation, Ahmad et al. focused on uncovering genes influenced by irradiation in *Zeugodacus cucurbitae* that are linked to critical developmental anomalies through comparative transcriptomics and subsequent targeted gene knockdown techniques. Their results provide strong evidence suggesting that irradiation can effectively identify candidate genes essential for developing future RNA interference (RNAi)-based strategies for pest control.

Light traps have been used to monitor and safeguard crops against insect pests for many years. As a crucial component of integrated pest management strategies, these traps have gained popularity in capturing crop-damaging insects while decreasing reliance on chemical pesticides. Recently, there has been significant interest in light trap technology as an environmentally friendly alternative to synthetic pesticide use. The current research conducted by Jiang et al. examined how factors such as duration of light exposure, gender differences, and circadian rhythms affect the phototactic behaviour of female and male *Dastarcus helophoroides* beetles. By employing various wavelengths of light, Jiang et al. analyzed gene expression patterns among females and males subjected to white light exposure. Findings indicated that the beetles exhibited negative phototaxis when exposed to light, suggesting a potential synergistic molecular network governing their response to illumination during nocturnal activity.

Insects are ectothermic organisms, and similar to other ectotherms, their survival, development, and reproduction can be significantly affected by exposure to temperature extremes. Recent research suggests that the threat posed by climate change to insect populations may be greater than earlier assessments have suggested (Johansson et al., 2020). Numerous insect species utilise phenotypic plasticity and genetic diversity to cope with these temperature fluctuations. Nevertheless, relying solely on these plasticity mechanisms is insufficient for many insect populations to withstand extreme temperatures. Consequently, further investigations using key insect models are essential to understanding how insects adapt to extreme thermal variations. In this series, there is one review on cold tolerance strategy and cryoprotectants, while there are two research articles covering heat stress and extracellular freezing responses. This study provides a theoretical basis for future research on the overwintering and potential distribution and related prediction of *Megabruchidius dorsalis* adults. In their review, Chen et al. examined the cold tolerance mechanisms of the adult seed beetle *M. dorsalis* alongside the effects of low temperatures on its physiological and biochemical processes. This research establishes a theoretical framework for future investigations into overwintering behaviours and potential distribution patterns of *M. dorsalis* adults.

Many species of insects have developed mechanisms to endure extracellular freezing, yet the fundamental principles underlying their natural freeze tolerance are not fully comprehended. Insects often use either freeze tolerance or freeze avoidance strategies; they maintain liquid bodily fluids while permitting ice formation in the extracellular spaces rather than within cellular interiors. Štětina and Koštál conducted a comparative analysis of mitochondrial structural and functional characteristics between larvae phenotypes that are sensitive to freezing and those that exhibit freeze tolerance in the drosophilid fly, *Chymomyza crostata*. Their research revealed that exposure to extracellular freezing triggered a permeability transition in the inner mitochondrial membrane.

*Cyrtorhinus lividipennis* (Reuter) is a hemipteran predator that targets the brown planthopper (BPH), *Nilaparvata lugens*, which poses a significant threat to rice crops. The effects of global warming are intensifying thermal stress, adversely affecting both the fitness and predatory abilities of *Cyrtorhinus lividipennis*. Consequently, it is crucial to explore how *C. lividipennis* responds to heat stress by identifying key resistance factors that can enhance its survival rates and improve its hunting efficiency under such conditions. Numerous studies have indicated that sphingolipids play a role in regulating responses to thermal stress. Ceramide-degrading enzymes (CDases) are vital metabolic enzymes involved in ceramide metabolism. Research conducted by Chen et al. identified two homologous CDase genes from genomic and transcriptomic databases related to *C. lividipennis*, uncovering potential regulatory mechanisms at play.

Under natural conditions, environmental stress encompasses a multifaceted array of abiotic and biotic factors, often leading organisms to encounter several stressors simultaneously. Investigating the co-variables that affect an organism’s reaction to multiple simultaneous stressors, or their combinations is crucial for understanding its threshold limits and homeostatic plasticity. This significant issue was explored by Bomble and Nath using *Drosophila melanogaster* as their model organism. Notably, all types of stressors triggered a shared oxidative stress response regardless of how they were administered. Their research demonstrated the production of reactive nitrogen species (RNS) alongside reactive oxygen species (ROS), establishing a connection between oxidative stress with desiccation, heat exposure, and starvation in *D. melanogaster* larvae. This study represents the first documentation of RONS (reactive oxygen and nitrogen species) generation following combined abiotic stresses in *D. melanogaster*, offering valuable physiologically relevant insights into these processes.

## Conclusion

Insects are vital components of ecosystems, and their resilience and adaptability will have significant implications for biodiversity, ecosystem services, and agricultural practices in a changing world. The stress responses and adaptations of insects in a changing environment are complex and multifaceted. As research in this area continues to evolve, it will be essential to integrate findings across disciplines to develop a comprehensive understanding of how insects will cope with ongoing environmental changes. This knowledge is vital for predicting ecological outcomes and informing conservation and agricultural practices.
